# Supplementary material for: On the origin of the extremely different solubilities of polyethers in water
Source: Nat Commun. 2019 Jun 28;10:2893. doi: 10.1038/s41467-019-10783-z (PMC6599002; doi:10.1038/s41467-019-10783-z)
Supplement: Supplementary file 1 — Supplementary Information [file 41467_2019_10783_MOESM1_ESM.pdf]

On the origin of the extremely different solubilities of polyethers in water

SUPPLEMENTARY INFORMATION

Bernd Ensing,[1] Martijn Tros,[1] Ambuj Tiwari,[1] Johannes Hunger,[2] Sérgio Rosa Domingos,[3] Cristóbal Pérez,[3] Gertien Smits,[4] Mischa Bonn,[2] Daniel Bonn,[5] and Sander Woutersen[1]

[1]*Van 't Hoff Institute for Molecular Sciences, University of Amsterdam, Science Park 904, 1098XH Amsterdam, The Netherlands*

[2]*Max Planck Institute for Polymer Research, Department of Molecular spectroscopy, Ackermannweg 10, 55128 Mainz, Germany*

[3]*Max Planck Institute for the Structure and Dynamics of Matter, Luruper Chaussee 149, 22761 Hamburg, Germany*

[4]*Swammerdam Institute for Life Sciences, University of Amsterdam, Science Park 904, 1098XH Amsterdam, The Netherlands*

[5]*Institute of Physics, University of Amsterdam, Science Park 904, 1098XH Amsterdam, The Netherlands*

(Dated: June 17, 2019)

SUPPLEMENTARY FIGURES

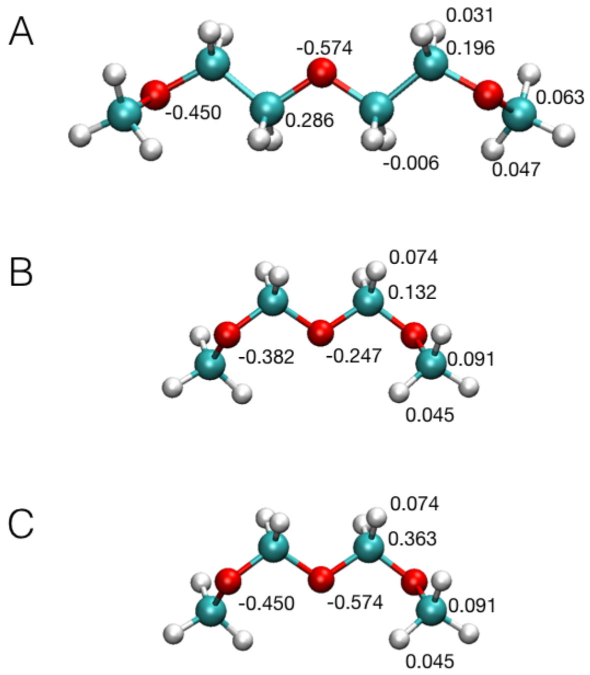

Supplementary Figure 1. Atomic partial charges used for the FF-MD simulation of the PEG3 molecule (panel A) and the POM3 molecule (panel B). These RESP charges were computed by fitting the electrostatic potential around each molecule, which was obtained from a quantum chemical HF/6-31G(d) calculation, following the GAFF forcefield generation procedure. We also created a fictitious POM3* molecule, carrying the oxygen charges of the PEG3 molecule, and with the central carbon charges adapted for total charge neutrality (panel C).

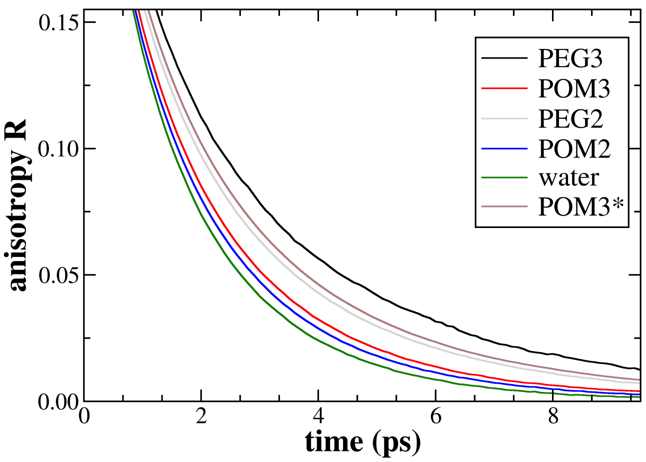

Supplementary Figure 2. O-H stretch anisotropy decay from FF-MD simulations of $x_{\mathrm{O}} = 0.12$ solutions of all PEG2, PEG3, POM2, POM3 and POM3* oligomers as compared to bulk water.

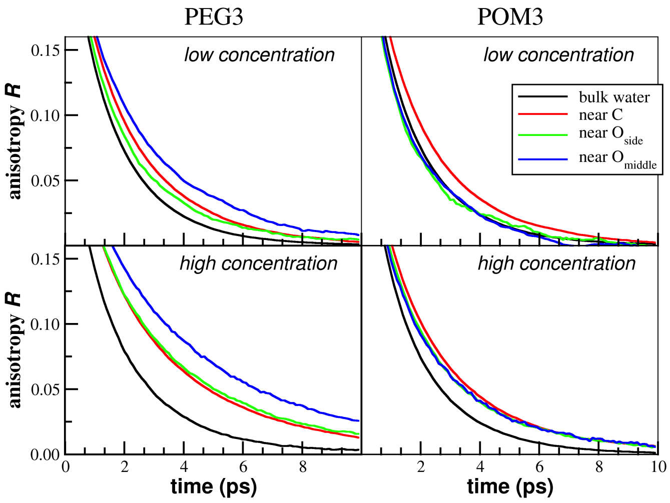

Supplementary Figure 3. O-H stretch anisotropy decay from FF-MD simulations of a single aqueous PEG3 molecule (top left panel) and POM3 molecule (top right) from water molecules near selected solute atoms. The bottom panels show the orientational time correlation in an $x_{\mathrm{O}} = 0.12$ solution.

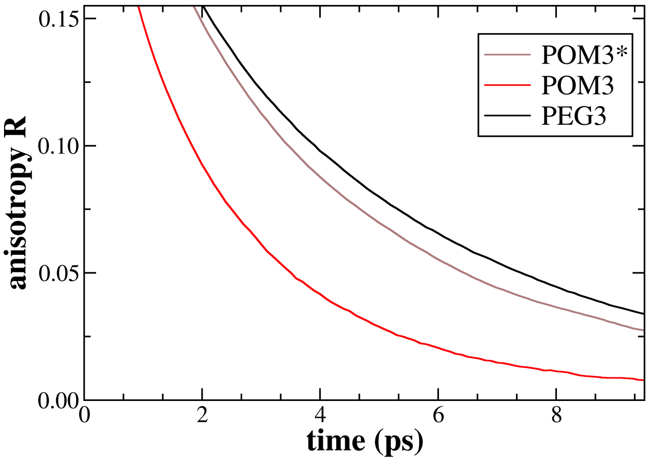

Supplementary Figure 4. O-H stretch anisotropy decay of water molecules around middle oxygen atoms of $x_{\mathrm{O}} = 0.12$ solutions of PEG3, POM3, and modified POM3* oligomers from FF-MD simulations.

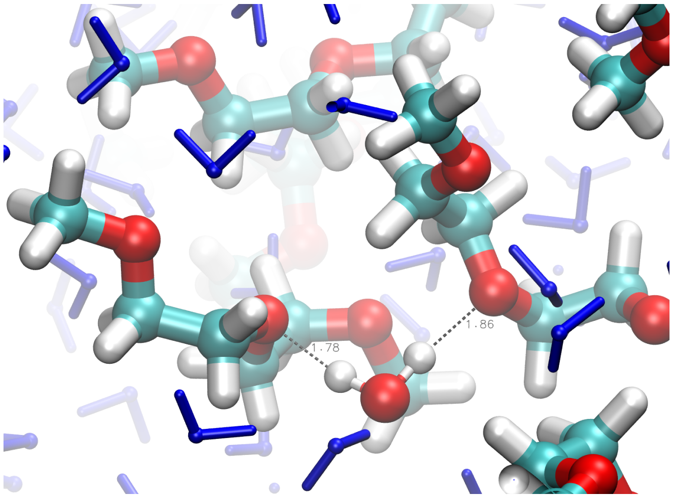

Supplementary Figure 5. Snapshot of a FF-MD simulation, showing a water molecule that forms a bridge between two PEG3 molecules.

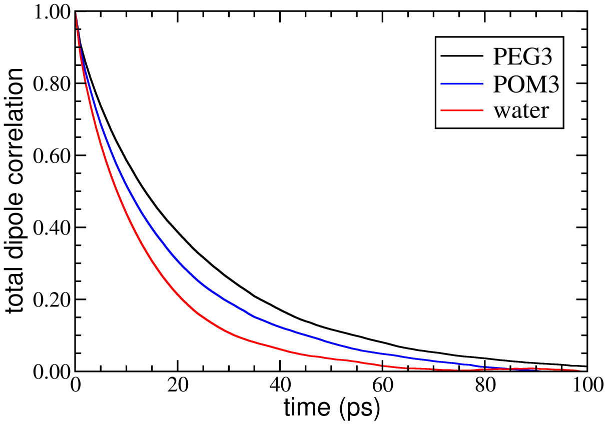

Supplementary Figure 6. Orientational correlation function of the total electric-dipole moment of PEG3 solution, POM3 solution (both with $x_{\mathrm{O}} = 0.12$), and neat water.

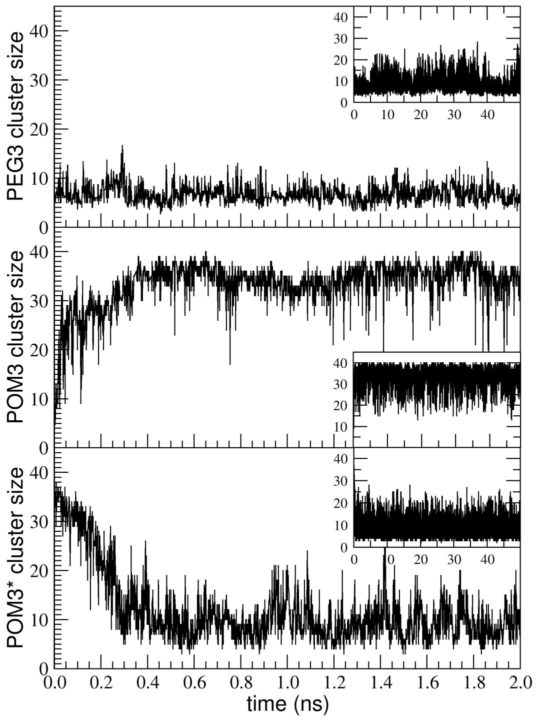

Supplementary Figure 7. Cluster size of the largest solute cluster, illustrating the perfect mixing of PEG3 with water (top panel) and the quick de-mixing of POM3 in water in less than a nanosecond of simulation time (middle panel). A configuration of a cluster taken from the POM simulation is seen to fully dissolve quickly, after we have replaced the POM molecules with the modified POM3* molecules carrying the PEG3 oxygen charges. The insets show the clustering behavior over the full 50 ns FF-MD simulation time.

Supplementary Figure 8. (Figure on next page) From top to bottom: snapshots at simulation times, $t = 0.01, 0.3, 0.5, 20$, and 50 ns, from the FF-MD simulations of aqueous PEG3 (right-hand-side panels), POM3, (middle), and the modified POM3* (right-hand-side), at the $x_O = 0.12$ concentration. Starting from equilibrated dispersed solute/solvent mixtures, POM3 is seen to aggregate in less than a nanosecond, whereas PEG3 remains fully mixed. The POM* species, carrying the PEG3 oxygen charges (see also Figure S1), quickly disperses when starting from an agglomerated configuration. In Figure 7, this (lack of) precipitation is quantified by plots of the largest cluster size.

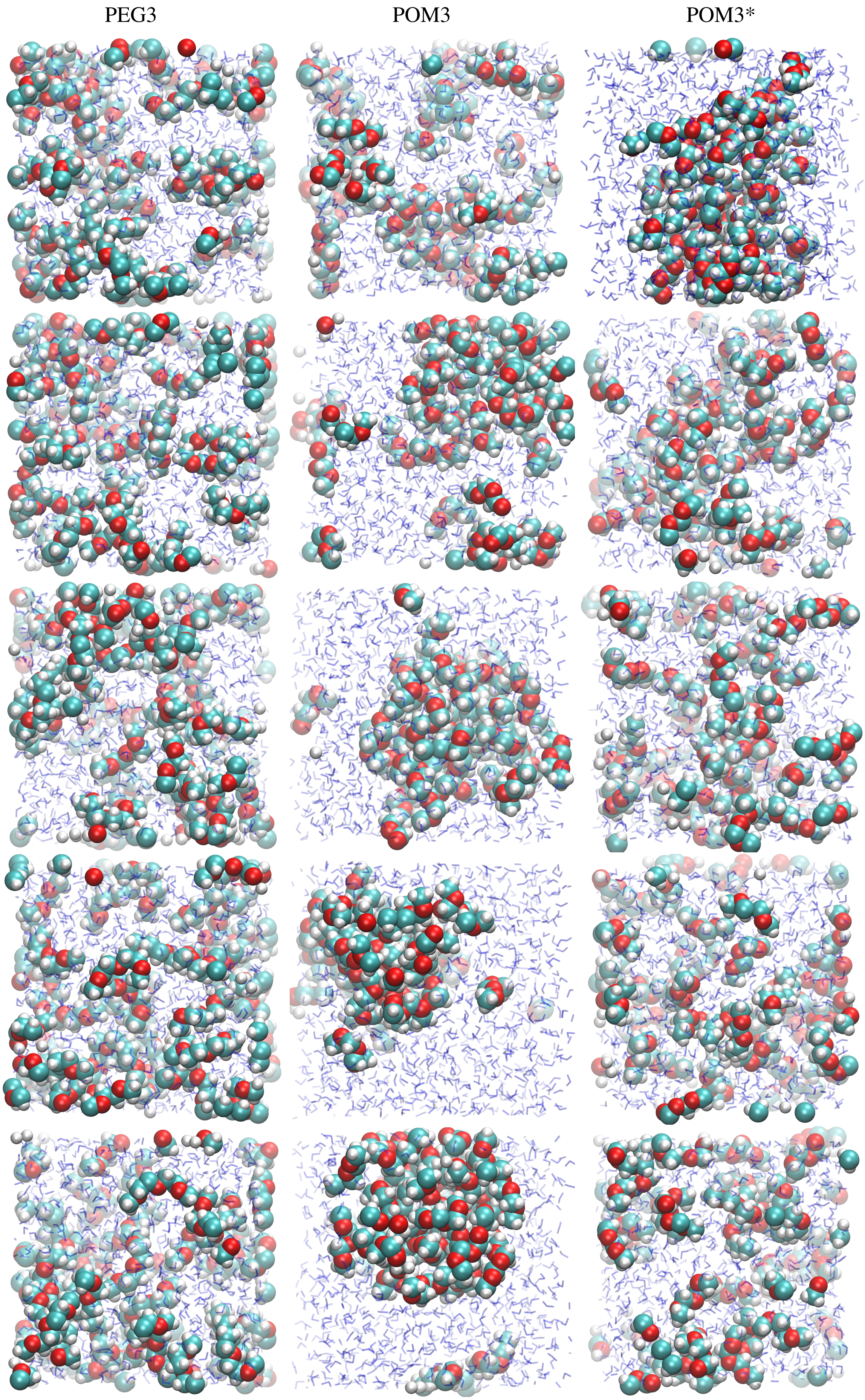
PEG3
POM3
POM3*

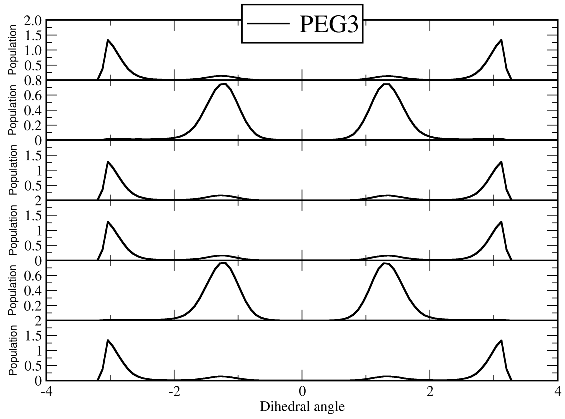

Supplementary Figure 9. Ensemble averages of the six sequential backbone dihedral angles in PEG3, which shows the most stable conformer to be TGTTGT (T=trans, G=Gauss).

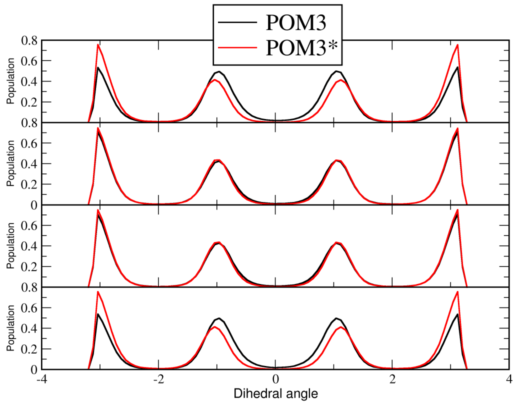

Supplementary Figure 10. Ensemble averages of the four sequential backbone dihedral angles in POM3, compared to those of the modified POM3*, which shows only minimal structural change upon change of the atomic charges.

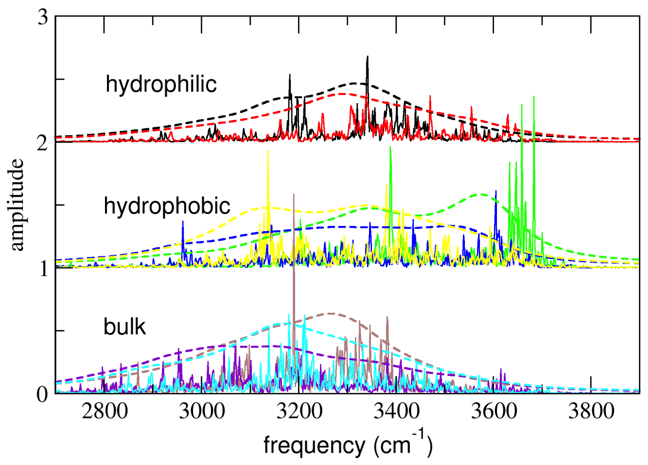

Supplementary Figure 11. Density of states spectra obtained from 10 ps DFT-MD simulations of a single PEG3 molecule in 120 water molecules, showing the spectra for two water molecules that form H-bonds to the PEG3 oxygen atoms (red and black), three selected water molecules in the hydrophobic part of the PEG3 coordination shell (blue, green, and yellow), and three bulk water molecules (brown, violet, cyan). The dashed lines show block averages calculated by convoluting the data with a Lorentzian with 80 cm^{-1} width. Despite the limited statistics, the spectra from both the hydrophilic and the hydrophobic regions are seen to be blue-shifted with respect to the bulk water solvent spectra.

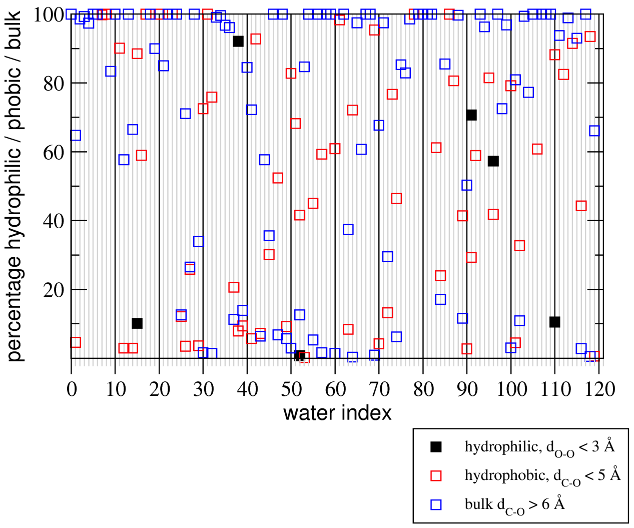

Supplementary Figure 12. Histogram showing for each of the 120 water molecules in the 10 ps DFT-MD simulation of a single PEG3 molecule in water the percentage of time spent in the hydrophilic region (black squares; $d(O-O) < 3$ Å), the hydrophobic region (red; $d(C-O) < 5$ Å), and the bulk region (blue; $d(C-O) > 6$ Å).

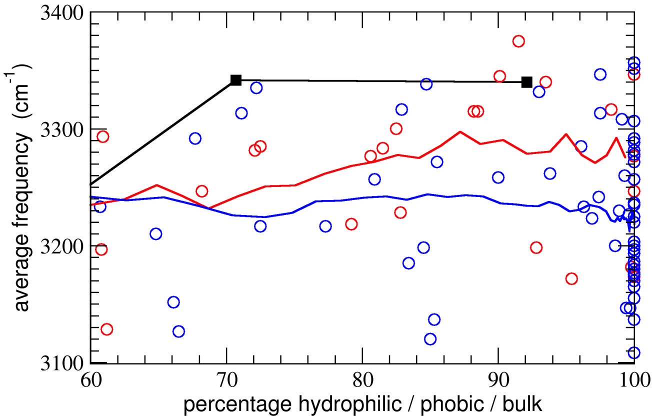

Supplementary Figure 13. The mean frequency (from the DoS spectra over the interval of 2750-4000 cm^{-1}) versus the percentage of time that each water molecule spent in the hydrophilic (black), hydrophobic (red), and bulk (blue) regions, during the 10 ps DFT-MD simulation. The running averages (black, red, and blue lines), show that the water molecules coordinating the PEG3 molecules have spectra that are blue shifted with respect to that of bulk water, irrespective of the hydrophilic or hydrophobic nature of the region.

SUPPLEMENTARY TABLES

Supplementary Table I. Fit parameters obtained from least-squares fits of eq. 1 to the observed vibrational anisotropy decays (data and fit curves shown in Fig. 1 of the main text). The values in parentheses indicate the uncertainty (2σ) in the last digit(s).

Solute	x_O	k_1/ps^{-1}	k_2/ps^{-1}	R_0
neat water		0.42(6)	2.3(4)	0.002(12)
dioxane	0.12	0.45(4)	3.1(7)	0.044(6)
dioxane	0.21	0.47(4)	2.9(7)	0.066(4)
trioxane	0.12	0.45(4)	3.4(4)	0.007(8)
dimethoxy-ethane	0.12	0.49(6)	2.9(4)	0.064(8)
dimethoxy-ethane	0.21	0.45(6)	2.6(8)	0.087(7)
dimethoxy-methane	0.12	0.43(5)	2.7(3)	0.009(9)
1,2-dimethoxypropane	0.12	0.56(5)	3.4(9)	0.075(5)
PEG-1000	0.12	0.63(6)	3.6(6)	0.075(5)

Supplementary Table II. System setups for the PEG3 and POM3 NVT simulations at the low and high concentrations, showing the (cubic) box sizes, and total numbers of solute and water molecules in each system, and the total simulation times, not counting the equilibration NPT and NVT parts. The POM3* systems contain the fictitious POM molecule with the modified atomic charges (see also Figure 1-C).

system	solute	water	box size (Å)	time (ns)
PEG2-LOW	1	494	24.74	50
POM2-LOW	1	417	23.37	50
PEG2-HIGH	60	880	33.27	50
POM2-HIGH	60	880	33.06	50
PEG3-LOW	1	717	27.99	50
POM3-LOW	1	490	24.64	50
POM3*-LOW	1	490	24.64	50
PEG3-HIGH	40	880	32.71	50
POM3-HIGH	40	880	32.14	50
POM3*-HIGH	40	880	32.19	50

Supplementary Table III. First three columns: average number of water molecules per solute molecule in each hydrophilic region (subdivided in middle solute oxygen and side solute oxygen) and hydrophobic region (denoted “carbon”). Divide by the number in parentheses to obtain the value per atom. Fourth and fifth row: average number of water molecules in the “bulk” region (not per solute molecule) and the total number of solvent molecules, respectively. The number of solute molecules in the “HC” system ($x_O = 0.12$) was 60 for POM2 and PEG2 and 40 for POM3 and PEG3 (see also Table II); the low concentration (“LC”) system contained a single solute molecule.

	Middle-O	Side-O	Carbon	Bulk	Total
PEG2 (LC)	-	2.21 (2)*	27.57 (4)	464.22	494
POM2 (LC)	-	1.79 (2)	24.62 (3)	390.59	417
PEG2 (HC)	-	1.51 (2)	8.37 (4)	287.38	880
POM2 (HC)	-	0.89 (2)	5.63 (3)	488.55	880
PEG3 (LC)	1.36 (1)	1.89 (2)	34.68 (6)	679.07	717
POM3 (LC)	0.62 (1)	1.87 (2)	28.61 (4)	458.90	490
PEG3 (HC)	1.20 (1)	1.49 (2)	14.18 (6)	205.04	880
POM3 (HC)	0.29 (1)	1.06 (2)	8.02 (4)	505.22	880

* The number of atoms of this type is given in parentheses.

SUPPLEMENTARY NOTES

Supplementary note 1. Vibrational-anisotropy fit results

The curves in Figure 1 of the main text are the results of a least-squares fit of a biexponential decay with an offset,

$$R(t) = a_1 \exp(-k_1 t) + a_2 \exp(-k_2 t) + R_0. \tag{1}$$

The first, main component is due to the orientational diffusion of the OD bonds. To obtain a good fit, we had to add a second, subpicosecond component that is due to inertial and librational motion of the OD groups[1] convoluted with our instrumental response of ∼200 fs FWHM. The residual anisotropy R_0 is due to water molecules that reorient on a time scale slower than our accessible delay range (determined by the lifetime of ∼1.5 ps of the vibrational excitation[2]). The fit parameters obtained from the least-squares fits are given in Table I. Note that the error bars represent lower limits for the uncertainty, since they do not take systematic contributions into account.

Supplementary note 2. MD Simulations

The classical *ab initio* (DFT-MD) and forcefield molecular dynamics (FF-MD) simulations carried out for this work, largely follow the same protocols as used in our recent study of water solvation of amphiphilic molecules[3].

To probe the structure and the dynamics of solvent water around the PEG and POM solute molecules at a highly accurate level of theory, DFT-MD simulations were performed of a single solute molecule of POM3 in aqueous solution and a single molecule of PEG3 in aqueous solution. These systems, containing 113 and 120 water molecules respectively, were first pre-equilibrated using NPT and NVT FF-MD simulations, as described hereafter, resulting in (cubic) box sizes of 15.396 Å and 15.754 Å, respectively. The first 3 ps of DFT-MD simulation were used for equilibration; the following 15 ps were used for statistical analysis, in particular for the calculation of intramolecular structural parameters (bond lengths, angles, dihedral angles), H-bond lengths, radial distribution functions, and vibrational spectra, much of which was used for benchmarking the FF-MD simulations. The DFT-MD simulation employs every MD step a quantum mechanical electronic structure calculation at the density functional theory (DFT) level of theory to obtain the

forces on the nuclei, which are used to propagate the atoms. The time step was 0.5 fs, and a csvr thermostat[4] with a time period of 500 fs maintained a constant temperature.

The DFT-MD simulations were computed with the Quickstep[5] module of the CP2K software[6], which uses a mixed Gaussian and plane-wave method[7] (GPW). We employed the PBE exchange correlation functional[8] , augmented with Grimme's D3 dispersion correction[9] to include Van der Waals interactions. The valence electron functions were expanded in a DZVP Gaussian basis[10] set and a plane wave expansion cutoff at 300 Ry. The nuclei and core electrons were modeled with the norm-conserving pseudopotentials of Goedecker et al[11].

To allow for simulations of larger systems and longer time scales, we have performed FF-MD simulations. FF-MD simulations of a single (POM3 or PEG3) solute molecule was dissolved in a large box with water to reproduce the water affinity and the slowdown effect on the solvent water molecules. These simulations were also verified against the *ab initio* molecular dynamics simulations to give qualitatively equivalent structural and dynamical properties for the aqueous oligomer solutions.

Next, FF-MD simulations of PEG3 and POM3 solutions were performed at the experimental concentration of $x_O = 0.12$ (referred to as "high polyether concentration" in the main text). Here, 40 solute molecules were dissolved in 880 water molecules in a cubic box subject to periodic boundary conditions. The Avogadro[12] and Packmol[13] programs were used to set up the initial configuration, which was relaxed and equilibrated in the NPT ensemble at $T = 300$ K and $p = 1$ atm for 1 ns followed by a 1 ns NVT simulation. The atoms were propagated using a four-level r-RESPA multi-timestep integrator[14], which is based on the velocity Verlet algorithm. The outer time step was set to 1 fs, with time steps of 0.5, 0.25, and 0.125 fs for the inner nested loops, in which the inter-molecular (*i.e.* Lennard-Jones Van der Waals cut-off at 10 Å and PPPM electrostatics) interactions are partitioned based on radius cut-offs (inner-to-middle: 4.5-6.0 Å and middle-to-outer: 8.0-10.0 Å). The intra-molecular (bond, angle, dihedral, improper, and one-four) interactions were evaluated in the inner loop. The Amber GAFF force field[15] together with the flexible TIP3P water model[16] was used, with atomic RESP charges obtained from quantum chemical calculations at the HF/6-31G(d) level of theory, following the standard GAFF procedure. For analysis, we performed 50 ns NVT production runs, saving positions every 1 ps. The temperature was controlled with a csvr thermostat with a period of 0.1 ps. The initial NPT simulations employed an isotropic Parrinello-Rahman barostat set to $p = 1$ bar, with a period of 1 ps and a Nose-Hoover chain thermostat set to $T = 300$ K, with a chain length of 4 and a period of 0.1 ps.

The MD simulations were performed with the LAMMPS package[17]. The box sizes, numbers of solute and solvent molecules, and simulation lengths of the production runs are compiled in Table II

Supplementary note 3. Anisotropy decay and water partitioning from FF-MD simulations

Figure 2 shows the O-H stretch anisotropy decay for all four polymers (PEG2, PEG3, POM2, POM3) modeled at the $x_{\mathrm{O}} = 0.12$ concentration as compared to pure water. The slowdown of the water rotation dynamics in the PEG oligomer solutions is evident from the comparison of the anisotropy decay with respect to the curves from the POM oligomer solutions. The slowdown is larger in the trimer solution (PEG3) compared to the dimer solution (PEG2).

By spatially partitioning the solvent, we can determine which water molecules cause the observed slowdown in PEG-like solutions. We distinguish between (1) water molecules (w) close to the solute (s) oxygens (distance $r_{\mathrm{H_w-O_s}} < 2.6$ Å), which we will refer to as "hydrophilic water", (2) other coordination shell water close to the solute carbon atoms ($r_{\mathrm{O_w-C_s}} < 5.0$ Å), termed "hydrophobic water", and (3) the remaining water, denoted "bulk". For the PEG3 and POM3 solutions, we also distinguish between hydrophilic water molecules close to the middle solute oxygen and molecules close to the outer solute oxygens. Figure 3 shows the anisotropy decays for diluted PEG3 (top left panel) and POM3 (top right panel). For the latter, the difference in rotational dynamics between first coordination shell waters and bulk water is very small, whereas in the PEG3 case, a clear slowdown is seen for the "hydrophilic waters" close to the central oxygen atom. Note that also the water molecule near the hydrophobic carbon (and hydrogen) atoms show some rotational slowdown with respect to bulk water, which is due to the excluded-volume effect that hydrophobic regions have on the number of hydrogen bond acceptor and donor sites around a water molecule.[18,19] At the high concentration, the difference in the decay of the first coordination shell water molecules and the "bulk" water is significantly larger, mainly because hydrating water molecules can interact simultaneously with two solute molecules, which causes a further slowdown (see also Figure 5 for an illustrative snapshot).

We also computed the O-H stretch anisotropy decay for the modified POM3* system (see Figure 1-C), which is shown in Figure 4. After modifying the oxygen charge, the solvent water molecules around the central POM3 oxygen show now a similar rotational slowdown as in the PEG3 system. The minor discrepancy can be attributed to the smaller hydrophobic (excluded-

volume) effect) due to the smaller number of neighboring carbon atoms in POM3* compared to PEG3 (see also the explanation in the main text).

Table III shows the average number of water molecules in the different "hydrophobic", "hydrophilic" and "bulk" regions around the PEG2, POM2, PEG3 and POM3 solute molecules for the low and high concentration ("LC" and "HC") FF-MD simulations. Finally, in Figure 5, we show a representative snapshot from a FF-MD simulations of a PEG3 solution, illustrating a solvating water molecule bridging between two neighboring solute molecules.

Supplementary note 4. Orientational relaxation of the total electric-dipole moment from FF-MD simulations

In the experiments we observe an interesting qualitative difference between the IR-anisotropy and dielectric-relaxation results: in solutions of POM-like polyethers the IR-anisotropy decay is essentially the same as that of neat water (main-text Fig. 2a,b), whereas the dielectric relaxation shows an increase of the time constant (main-text Fig. 3), albeit a significantly smaller increase than in PEG-type solutions. As discussed in the main text, the IR-anisotropy and dielectric-relaxation measurements on liquid water and aqueous solutions cannot be compared directly, because different rotational motions of the water are probed in these two types of experiments (rotation of an OH bond in the IR-anisotropy, and of the electric-dipole moment in the dielectric-relaxation measurements).[20] Another fundamental difference between the two types of measurements is that dielectric-relaxation is sensitive to correlation between orientational motion of the water molecules, whereas the IR anisotropy is not.[21,22]

We performed FF-MD simulations to investigate the qualitative difference in the IR and anisotropy measurements in more detail. For this purpose, we calculated the correlation function of the total electric-dipole moment of the PEG3 and POM3 solutions (the dielectric spectrum is essentially the Fourier transform of this correlation function[23]). In Figure 6, we plot the correlation function $\langle \vec{M}(t) \cdot \vec{M}(0) \rangle$ of the total electric-dipole moment $\vec{M}(t) = \sum_i \vec{\mu}_i(t)$, where $\vec{\mu}_i(t)$ is the electric-dipole moment vector of water molecule i, and the sum runs over all water molecules in the simulated box. We observe the same qualitative difference in the simulations as in the experiment: whereas the orientational OH-correlation function of POM3 solution is similar to that of neat water (Fig. 2), the dielectric response (Fig. 6) slows down significantly compared to neat water, by an amount of about half that observed in the PEG3 solution.

Supplementary note 5. Solubility seen from clustering of the polyether oligomers in FF-MD simulations

From the FF-MD simulations, the different solubilities of PEG3 and POM3 is immediately evident from the observation that the latter aggregates spontaneously in aqueous solution at a concentration of $x = 0.12$, whereas the PEG3 solution does not phase separate. The snapshots in Figure 8 show that the, initially well-dispersed, POM3 aggregates into small clusters on a time scale of hundreds of's picoseconds, forming a single stable cluster (with one or two remaining solitary molecules) in about a nanosecond. The PEG3 solution, on the other hand, remains well-mixed during the 50 ns simulation.

After about 1 nanosecond from the moment of cluster formation in the POM3 solution, we took a configuration and replaced the POM3 oxygen charges with the PEG3 oxygen charges, and adapted the central carbon charges to maintain overall charge neutrality (see also Figure 1). Snapshots from the following 50 ns FF-MD simulation of this fictitious POM3* solution are shown in the right-hand-side panels in Figure 8. The cluster dissolves in less than a nanosecond, confirming our hypothesis that the difference in charge distribution in the POM and PEG polyethers explains their remarkably different and counter-intuitive solubilities.

For a more quantitative analysis, we performed a clustering of configurations based on a 3 Å distance cut-off between oxygen atoms, using the"depth-first search" (DFS) clustering algorithm, as implemented in the PLUMED software[24]. In Figure 7, we plot the size of the largest cluster, measured by the number of solute molecules, for the PEG3, POM3, and modified POM3* solutions. The top panel shows that the largest "cluster" in the PEG3 solution contains on average about 10 molecules, but can contain over 20 molecules. However, visual inspection of the trajectory reveals that the solute molecules are mainly situated in head-to-tail configurations and remain fully hydrated by a coordination shell of solvent water. Instead, POM3 is seen to form a large aggregate containing near all (40) solute molecules within about 0.5 ns (middle panel). After modifying the POM3 oxygen charges as explained above (see Figure 1), the largest cluster quickly falls apart as the POM3* molecules dissolve in less than 0.5 ns.

Supplementary note 6. Effect of the oxygen charge on the oligomer structure

To test whether the change of the charge of the ether oxygen atom changes the structure of the oligomer, causing a change in solubility through a structural change, we computed the average dihedral angles along the oligomer chains from the FF-MD simulations of the concentrated PEG, POM and modified POM* systems. It is known that PEG can have different conformations, of which only the trans-gauss-trans-trans-gauss-trans (TGTTGT) conformation is found to be soluble[25–27]. The results are shown in Figure 9 for the PEG3 simulation and in Figure 10 for the POM3 and POM3* simulations. The former shows that the PEG3 molecules indeed adopt the TGT conformation, whereas the latter shows that modification of the atomic charges has only very little impact on the structural conformation.

Supplementary note 7. Blue shift of the OH stretch vibration of coordinating water molecules

The IR spectra of the O-H stretch vibration of water molecules in the solutions containing PEG-like molecules show a small blue-shift with respect to the pure water spectra, as shown in the insets of Figure 2 in the main text. This may seem to contradict the idea that PEG3 is strongly hydrated by water molecules, which should give rise to a population of red-shifted O-H stretch vibrations. To verify if hydrating water molecules around PEG oligomers indeed exhibit a lower O-H stretch frequency, we computed the vibrational spectra from additional DFT-MD simulation of a single PEG3 molecule in 120 water molecules. We saved the velocities of the atoms every 2 fs during the 10 ps simulation, and computed for each water molecule the density of states (i.e. the vibration spectrum) as the Fourier transform of the velocity auto-correlation function.

Figure 11 shows these spectra for two "hydrophilic" water molecules that formed hydrogen bonds to the ether oxygens during most of the simulation and for three "hydrophobic" water molecules that were found in the vicinity of the carbon atoms. These spectra are compared to those of three "bulk" water molecules. The spectra of both the hydrophilic and the hydrophobic water molecules are blue-shifted with respect to that of the bulk water molecules.

Figure 12 shows the same result as the mean O-H stretch frequency averaged over all hydrophilic, hydrophobic, and bulk water molecules, as a function of the percentage of time that a water molecule spends in each region. For water molecules that are 100% of the simulation time in a certain region, the coordinating molecules are again seen to be blue shifted. We note that in

our short DFT-MD simulation, only 2 water molecules were seen to remain H-bonded (within our somewhat strict definition of the O-O distance being shorter than 3 Å) for more that 60% of the simulation time, which limits the statistics.

[1] Fecko, C. J., Loparo, J. J., Roberts, S. T. & Tokmakoff, A. Local hydrogen bonding dynamics and collective reorganization in water: Ultrafast infrared spectroscopy of HOD/D_2O. *J. Chem. Phys.* **122**, 054506 (2005).

[2] Rezus, Y. L. A. & Bakker, H. J. On the orientational relaxation of HDO in liquid water. *J. Chem. Phys.* **123**, 114502 (2006).

[3] Brandeburgo, W. H., van der Post, S. T., Meijer, E. J. & Ensing, B. On the slowdown mechanism of water dynamics around small amphiphiles. *Physical Chemistry Chemical Physics* **17**, 24968–24977 (2015).

[4] Bussi, G., Donadio, D. & Parrinello, M. Canonical sampling through velocity rescaling. *J. Comp. Phys.* **126**, 014101 (2007).

[5] VandeVondele, J. *et al.* Quickstep: Fast and accurate density functional calculations using a mixed gaussian and plane waves approach. *Comput. Phys. Comm.* **167**, 103–128 (2005).

[6] The CP2K Developers Group. `http://www.cp2k.org`.

[7] Lippert, G., Hutter, J. & Parrinello, M. A hybrid gaussian and plane wave density functional scheme. *Mol. Phys.* **92**, 477–488 (1997).

[8] J. P. Perdew and K. Burke and M. Ernzerhof. Generalized Gradient Approximation Made Simple. *Phys. Rev. Lett.* **77**, 3865–3868 (1996).

[9] Stefan Grimme and Jens Antony and Stephan Ehrlich and Helge Krieg. A Consistent And Accurate Ab Initio Parametrization Of Density Functional Dispersion Correction (Dft-D) For The 94 Elements H-Pu. *J. Comp. Phys.* **132**, 154104 (2010).

[10] VandeVondele, J. & Hutter, J. Gaussian basis sets for accurate calculations on molecular systems in gas and condensed phases. *The Journal of chemical physics* **127**, 114105 (2007).

[11] Goedecker, S., Teter, M. & Hutter, J. Separable dual-space gaussian pseudopotentials. *Phys. Rev. B* **54**, 1703 (1996).

[12] Hanwell, M. D. *et al.* Avogadro: an advanced semantic chemical editor, visualization, and analysis platform. *Journal of cheminformatics* **4**, 17 (2012).

[13] Martínez, L., Andrade, R., Birgin, E. G. & Martínez, J. M. Packmol: a package for building initial configurations for molecular dynamics simulations. *Journal of computational chemistry* **30**, 2157–2164 (2009).

[14] Tuckerman, M., Berne, B. J. & Martyna, G. J. Reversible multiple time scale molecular dynamics. *J.*

Comp. Phys. **97**, 1990 (1992).

[15] Wang, J., Wolf, R. M., Caldwell, J. W., Kollman, P. A. & Case, D. A. Development and testing of a general amber force field. *Journal of computational chemistry* **25**, 1157–1174 (2004).

[16] Jorgensen, W. L., Chandrasekhar, J., Madura, J. D., Impey, R. W. & Klein, M. L. Comparison of simple potential functions for simulating liquid water. *The Journal of chemical physics* **79**, 926–935 (1983).

[17] Plimpton, S. Fast parallel algorithms for short-range molecular dynamics. *Journal of computational physics* **117**, 1–19 (1995).

[18] Laage, D. & Hynes, J. T. A molecular jump mechanism of water reorientation. *Science* **311**, 832–835 (2006).

[19] Laage, D., Stirnemann, G., Sterpone, F. & Hynes, J. T. Water jump reorientation: from theoretical prediction to experimental observation. *Acc. Chem. Res.* **45**, 53–62 (2012).

[20] Tielrooij, K. J., Garcia-Araez, N., Bonn, M. & Bakker, H. J. Cooperativity in ion hydration. *Science* **328**, 1006–1009 (2010).

[21] Van der Loop, T. H. *et al.* Structure and dynamics of water in nonionic reverse micelles: A combined time-resolved infrared and small angle x-ray scattering study. *J. Chem. Phys.* **137**, 044503 (2012).

[22] Van der Loop, T. H. *et al.* Structure and dynamics of water in nanoscopic spheres and tubes. *J. Chem. Phys.* **141**, 18C535 (2014).

[23] Kubo, R., Toda, M. & Hashitsume, N. *Statistical Physics II. Nonequilibrium Statistical Mechanics* (Springer, Berlin, 1985).

[24] Tribello, G. A., Bonomi, M., Branduardi, D., Camilloni, C. & Bussi, G. Plumed 2: New feathers for an old bird. *Computer Physics Communications* **185**, 604–613 (2014).

[25] Begum, R. & Matsuura, H. Conformational properties of short poly (oxyethylene) chains in water studied by ir spectroscopy. *Journal of the Chemical Society, Faraday Transactions* **93**, 3839–3848 (1997).

[26] Goutev, N., Ohno, K. & Matsuura, H. Raman spectroscopic study on the conformation of 1, 2-dimethoxyethane in the liquid phase and in aqueous solutions. *The Journal of Physical Chemistry A* **104**, 9226–9232 (2000).

[27] Wahab, S. A., Harada, T., Matsubara, T. & Aida, M. Quantum chemical study of the interaction of the short-chain poly (oxyethylene) s ch3 (och2ch2) m och3 (c1e m c1; m= 1 and 2) with a water molecule in the gas phase and in solutions. *The Journal of Physical Chemistry A* **110**, 1052–1059 (2006).
